# Predictors of HIV rebound differ by timing of antiretroviral therapy initiation

**DOI:** 10.1172/jci.insight.173864

**Published:** 2024-02-08

**Authors:** Jonathan Z. Li, Meghan Melberg, Autumn Kittilson, Mohamed Abdel-Mohsen, Yijia Li, Evgenia Aga, Ronald J. Bosch, Elizabeth R. Wonderlich, Jennifer Kinslow, Leila B. Giron, Clara Di Germanio, Mark Pilkinton, Lynsay MacLaren, Michael Keefer, Lawrence Fox, Liz Barr, Edward Acosta, Jintanat Ananworanich, Robert Coombs, John Mellors, Steven Deeks, Rajesh T. Gandhi, Michael Busch, Alan Landay, Bernard Macatangay, Davey M. Smith, for the AIDS Clinical Trials Group A5345 Study Team

**Affiliations:** 1 Brigham and Women’s Hospital, Harvard Medical School, Boston, Massachusetts, USA.; 2 The Wistar Institute, Philadelphia, Pennsylvania, USA.; 3 University of Pittsburgh, Pittsburgh, Pennsylvania, USA.; 4 Harvard T.H. Chan School of Public Health, Boston, Massachusetts, USA.; 5 Southern Research, Frederick, Maryland, USA.; 6 Rush University Medical Center, Chicago, Illinois, USA.; 7 University of California, San Francisco, San Francisco, California, USA.; 8 Vitalant Research Institute, San Francisco, California, USA.; 9 Vanderbilt University Medical Center, Nashville, Tennessee, USA.; 10 Whitman Walker Health, Washington, DC, USA.; 11 University of Rochester, Rochester, New York, USA.; 12 National Institute of Allergy and Infectious Diseases, NIH, Rockville, Maryland, USA.; 13 AIDS Clinical Trials Group Community Scientific Subcommittee, Los Angeles, California, USA.; 14 University of Alabama, Birmingham, Alabama, USA.; 15 Thai Red Cross AIDS Research Centre, Bangkok, Thailand.; 16 University of Washington, Seattle, Washington, USA.; 17 Massachusetts General Hospital, Harvard Medical School, Boston, Massachusetts, USA.; 18 University of California, San Diego, San Diego, California, USA.; 19 The AIDS Clinical Trials Group A5345 Study Team is detailed in Supplemental Acknowledgments.

**Keywords:** AIDS/HIV, AIDS vaccine, Adaptive immunity

## Abstract

**BACKGROUND:**

Identifying factors that predict the timing of HIV rebound after treatment interruption will be crucial for designing and evaluating interventions for HIV remission.

**METHODS:**

We performed a broad evaluation of viral and immune factors that predict viral rebound (AIDS Clinical Trials Group A5345). Participants initiated antiretroviral therapy (ART) during chronic (*N* = 33) or early (*N* = 12) HIV infection with ≥ 2 years of suppressive ART and restarted ART if they had 2 viral loads ≥ 1,000 copies/mL after treatment interruption.

**RESULTS:**

Compared with chronic-treated participants, early-treated individuals had smaller and fewer transcriptionally active HIV reservoirs. A higher percentage of HIV Gag-specific CD8^+^ T cell cytotoxic response was associated with lower intact proviral DNA. Predictors of HIV rebound timing differed between early- versus chronic-treated participants, as the strongest reservoir predictor of time to HIV rebound was level of residual viremia in early-treated participants and intact DNA level in chronic-treated individuals. We also identified distinct sets of pre–treatment interruption viral, immune, and inflammatory markers that differentiated participants who had rapid versus slow rebound.

**CONCLUSION:**

The results provide an in-depth overview of the complex interplay of viral, immunologic, and inflammatory predictors of viral rebound and demonstrate that the timing of ART initiation modifies the features of rapid and slow viral rebound.

**TRIAL REGISTRATION:**

ClinicalTrials.gov NCT03001128

**FUNDING:**

NIH National Institute of Allergy and Infectious Diseases, Merck

## Abstract 2

<p>Evaluation of the&nbsp;AIDS Clinical Trial Group A5345 identified&nbsp;immune and inflammatory markers that differentiate&nbsp;individuals with rapid versus slow HIV rebound after treatment interruption.</p>

## Introduction

Therapeutics to achieve antiretroviral therapy–free (ART-free) HIV remission will require eventual validation in treatment interruption trials, but the risks, expense, and required duration of the treatment interruptions are obstacles for their use in early-phase clinical studies. As the HIV field increasingly turns toward developing therapies to achieve sustained ART-free HIV remission, biomarkers that can reliably predict this outcome are urgently needed. Identifying biomarkers that predict time to HIV rebound after treatment interruption would be useful to prioritize promising treatment strategies for further clinical development and to avoid the cost and risk of treatment interruption studies for therapies that are unlikely to result in a delay of viral rebound (1, 2). The discovery of virologic, immunologic, and other host biomarkers that can predict delayed HIV rebound after treatment interruption could streamline the allocation of limited resources and avoid the exposure of individuals to ineffectual therapies and ART interruption. Such biomarkers could also identify novel targets for intervention aimed at achieving ART-free remission.

HIV rebound generally occurs within 3–4 weeks after ART discontinuation (3, 4). Prior studies have reported that certain factors may influence the timing of HIV rebound, including early initiation of ART and the size of the total and expressed HIV reservoir (3, 5–7). These findings provide important proof of principle, but a systematic evaluation is needed across a broad range of viral, immune, and host biomarkers that may predict delayed viral rebound after treatment interruption.

The AIDS Clinical Trials Group (ACTG) trial A5345 was a prospective treatment interruption study to identify biomarker predictors of HIV rebound timing. Participants treated during either early or chronic HIV infection and with virologic suppression were enrolled and underwent an intensively monitored treatment interruption. We have previously reported the viral load kinetics for the participants of A5345, showing that modern ART regimens had minimal impact on the timing of HIV rebound after ART interruption and that early ART initiation was associated with a significant delay in time to HIV rebound (8). Using stored pre–treatment interruption specimens, we sought to identify viral, immune, and host predictors of time to HIV rebound that could be crucial for designing and evaluating interventions aimed at inducing HIV remission without ART.

## Results

The analysis population includes 45 participants who initiated treatment interruption and are in the evaluable population. This includes 33 individuals who initiated ART during chronic infection (chronic treated) and 12 participants who initiated ART during early infection (early treated). The demographics of the chronic- and early-treated groups were generally comparable, although race and pre-ART viral loads were different (Supplemental Table 2; supplemental material available online with this article; https://doi.org/10.1172/jci.insight.173864DS1). For the time-to-rebound analysis, we used as the primary outcome the time to confirmed viral load ≥ 1,000 HIV RNA copies/mL. The first viral load ≥ 1,000 copies/mL was used as the day of rebound. The median time to viral rebound was 22 days among all A5345 participants (range 13 to 230 days). The median duration of ART was 4 years in the early-treated participants and 10 years in the chronic-treated participants. The duration of ART was not significantly associated with time to viral rebound.

**Figure 1:**
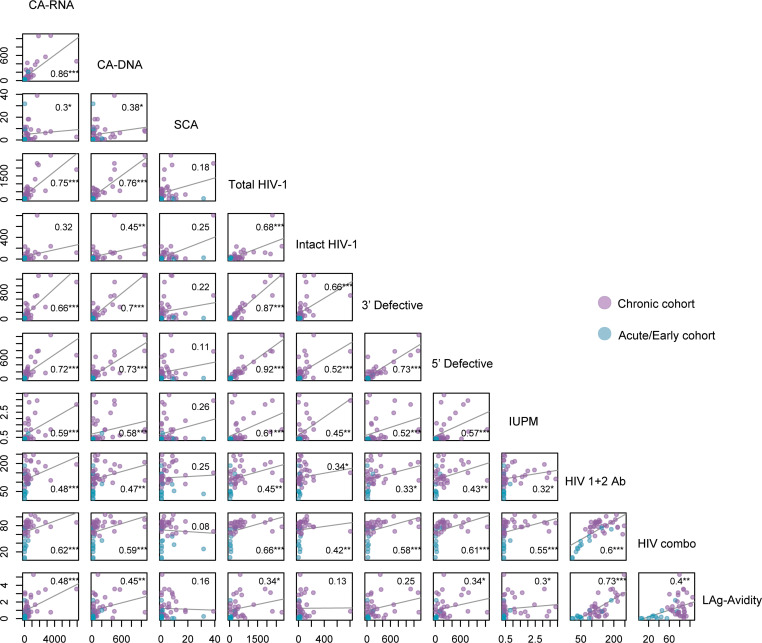
Relationship between HIV reservoir measures and antibody levels before treatment interruption. Correlation plots with Spearman rho values. ***P < 0.001, **P < 0.01, *P < 0.05. CA-RNA, unspliced cell-associated RNA; CA-DNA, total HIV proviral DNA; SCA, integrase single-copy assay; Total HIV-1, total HIV proviral DNA by the intact proviral DNA assay (IPDA); IUPM, infectious units per million resting CD4^+^ cells by the differentiation quantitative viral outgrowth assay; LAg-Avidity, HIV-1 limiting antigen avidity enzyme immunoassay.

After study entry, all participants provided samples via leukapheresis for baseline (on-ART) reservoir and immunology studies. In a combined analysis of all A5345 participants, significant associations were detected at baseline between the various reservoir measurements, as described below. Notably, levels of intracellular unspliced HIV RNA transcripts (cell-associated RNA, CA-RNA) correlated more strongly with total and defective proviral DNA levels than levels of intact proviral DNA (Spearman *r* = 0.32, *P* = NS, Figure 1). This was the case with total proviral DNA measured by the traditional quantitative PCR measure of total DNA (CA-DNA, *r* = 0.86, *P* < 0.001) or by the IPDA (total HIV DNA, *r* = 0.75, *P* < 0.001) and with either 3′ defective (*r* = 0.66, *P* < 0.001) or 5′ defective (*r* = 0.72, *P* < 0.001) proviral levels as measured by the IPDA. These results highlight the transcriptional activity of the defective proviral reservoir and its preponderance among measured transcripts. IUPM values were significantly correlated with multiple measures of the HIV reservoir, including IPD (*r* = 0.45, *P* < 0.01). In contrast, iSCA levels had the weakest association with other reservoir measures and were modestly correlated only with CA-RNA and CA-DNA (Figure 1). Significant positive correlations were also detected between nearly all HIV reservoir markers and 3 different HIV antibody levels (HIV 1+2 Ab, HIV combo Ab, and LAg avidity tests), demonstrating that HIV-specific antibody levels largely reflect the size and activity of the HIV reservoir, as previously seen (9). The only reservoir measure that was not significantly associated with any of the antibody levels was residual viremia as measured by the iSCA.

**Figure 2:**
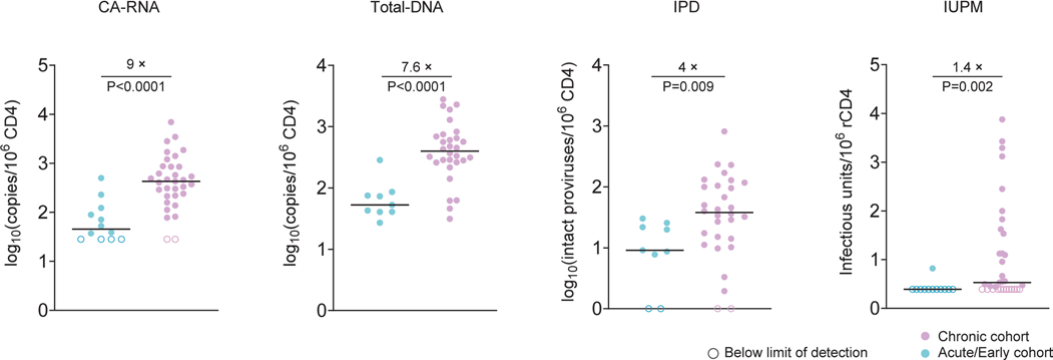
HIV reservoir comparison between early- and chronic-treated participants. Unspliced cell-associated HIV RNA, total HIV DNA by the intact proviral DNA assay (IPDA), intact proviral DNA (IPD) by the IPDA, and infectious units per million resting CD4^+^ cells (IUPM) by the differentiation quantitative viral outgrowth assay (dQVOA). Open circles represent values that are below the limit of quantification. Median lines and fold-change between early- and chronic-treated participants are shown. *P* values by the Wilcoxon rank sum test.

Early-treated participants had smaller and less transcriptionally active HIV reservoirs versus chronic-treated participants, as reflected by significantly lower levels of CA-RNA (9-fold difference in median values, *P* < 0.001, Figure 2), total DNA by the IPDA (7.6-fold, *P* < 0.001), intact proviral DNA (4-fold, *P* = 0.009), and IUPM by the dQVOA (1.4-fold, *P* = 0.002). A substantial subset of IUPM data was below the limit of quantification, and this fold-difference is likely an underestimate. Early-treated participants also had a 2.6-fold higher percentage of intact proviral DNA, though this was not statistically significant.

We next assessed the ability of HIV reservoir markers at the pre–treatment interruption time point to predict the timing of HIV rebound among all participants of A5345. One of the primary prespecified objectives of the study was to evaluate the relationship between the size of the transcriptionally active HIV reservoir, as measured by CA-RNA, and timing of HIV rebound. Among all participants, we found that higher CA-RNA levels were modestly associated with more rapid HIV rebound (CA-RNA: *r* = –0.26, *P* = 0.08; Table 1). Similar modest negative associations were also seen with the other measures of HIV reservoir size and activity. Notably, the strength of this relationship varied by the timing of ART initiation. The strongest reservoir predictor of faster time to HIV rebound was higher level of intact proviral DNA in chronic-treated participants (*r* = –0.37, *P* = 0.04) and higher level of iSCA in early-treated participants (*r* = –0.68, *P* = 0.02).

**Figure 3:**
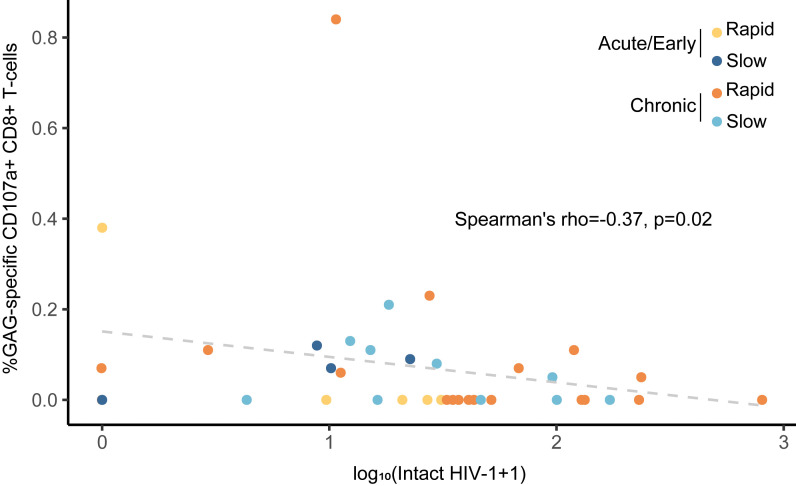
Higher percentage of HIV Gag-specific CD107a^+^ CD8^+^ T cells is associated with lower intact HIV-1 DNA levels. Data points are color-coded by timing of ART initiation and rapid versus slow viral rebound. Rapid rebound is defined as meeting ART restart criteria < 4 weeks, and slow rebound is defined as meeting ART restart criteria on or after week 4.

**Figure 4:**
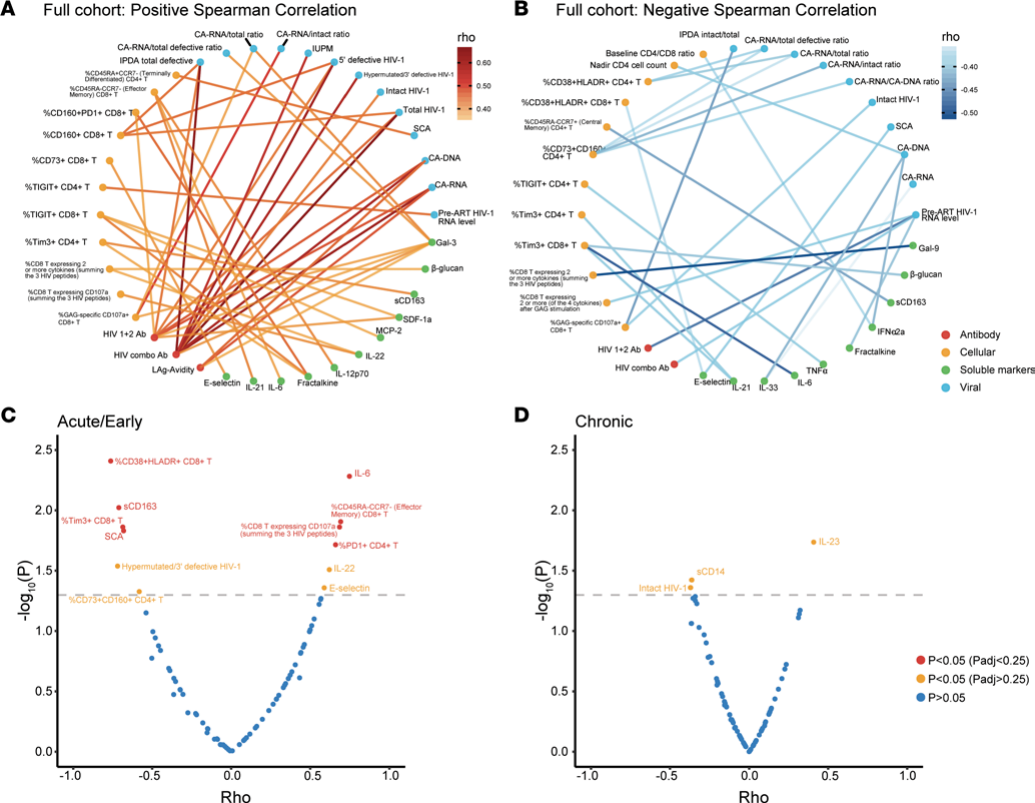
Correlation of reservoir, immune, and inflammatory markers with timing of HIV rebound. (**A** and **B**) Correlation map with lines connecting factors between categories (antibody, cellular, soluble inflammatory markers, and viral) meeting either (**A**) Spearman *r* > 0.35 and unadjusted *P* < 0.05 or (**B**) *r* < –0.35 and unadjusted *P* < 0.05. (**C** and **D**) Volcano plot of Spearman correlations with timing of HIV rebound in (**C**) acute/early-treated and (**D**) chronic-treated participants. Factors associated with earlier rebound are on the left and factors associated with delayed rebound on the right. Padj values were adjusted *P* values using the Benjamini-Hochberg method, and those with Padj < 0.25 are included given the exploratory nature of this analysis.

We also evaluated the frequencies of T cell maturation subsets, levels of T cell activation (HLA-DR^+^CD38^+^) and exhaustion (PD-1, TIM3, TIGIT, LAG3, and CD160), and HIV-specific T cell polyfunctional responses (CD107a, TNF-α, IL-2, and IFN-γ) to Gag, Pol, and Env peptide pools, as well as several plasma markers of inflammation and immune activation. There were no consistent differences in the immune parameters between early- and chronic-treated participants (Supplemental Table 3). Among all participants, a higher percentage of HIV Gag-specific CD8^+^ T cell cytotoxic response (CD107a) was associated with a lower intact proviral DNA level (*r* = –0.37, *P* = 0.02, Figure 3 and Figure 4B). While HIV-specific antibody levels were closely correlated with multiple reservoir measures, there were relatively few relationships between HIV-specific T cell responses and HIV reservoir markers (Figure 4, A and B). In early-treated participants, a higher percentage of CD8^+^ T cells expressing ≥ 2 effector cytokines was also associated with lower intact proviral DNA levels (*r* = –0.66, *P* = 0.05). Overall, there were strong correlations for assays within each of the reservoir, immunology, and inflammatory marker categories, but there were relatively few correlations between categories (Figure 4A). Supplemental Figure 1 shows examples of significant correlations between viral, immune, and inflammatory markers.

Distinct sets of viral, immune, and inflammatory markers associated with timing of viral rebound were detected between those initiating ART during early and chronic infection. In the early-treated cohort, higher levels of several pre–treatment interruption markers were correlated with more rapid viral rebound, including virologic (iSCA, 3′ defective proviruses), immunologic (%CD38^+^HLA-DR^+^CD8^+^ T cells, %TIM3^+^CD8^+^ T cells, %CD73^+^CD160^+^CD4^+^ T cells), and inflammatory (soluble CD163, sCD163). Factors associated with a slower viral rebound included immunologic (%CD45RA^–^CCR7^–^ effector memory CD8^+^ T cells, %PD-1^+^CD4^+^ T cells, and %HIV-specific CD8^+^ T cells expressing CD107a) and inflammatory (IL-6 and IL-22) (Figure 4C). In the chronic-treated cohort, only levels of intact proviral DNA and sCD14 were associated with more rapid viral rebound, and IL-23 levels were associated with slower rebound (Figure 4D).

**Figure 5:**
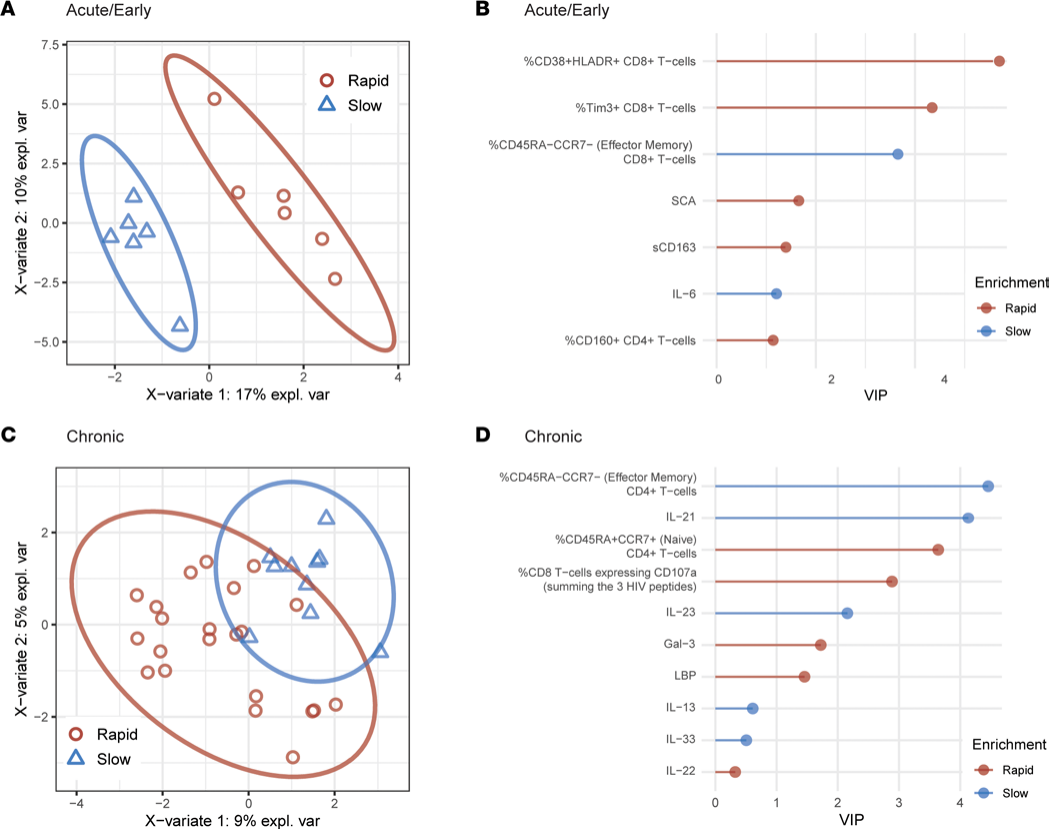
Viral and immune predictors of HIV rebound timing. Sparse variant partial least squares discriminant analysis (sPLS-DA) of individuals with rapid (< 4 weeks) or slow (≥ 4 weeks) viral rebound. PLS-DA score derived from features after sPLS-DA feature down-selection, demonstrating distinction between rapid and slow viral rebound groups in (**A**) acute/early-treated and (**C**) chronic-treated participants. Variable importance in projection (VIP) scores for selected features in (**B**) acute/early-treated and (**D**) chronic-treated participants. expl. var, explained variance.

In the multivariate partial least squares discriminant analysis, we identified distinct sets of viral, immunologic, and inflammatory biomarkers that could differentiate between rapid (< 4 weeks) and slow (≥ 4 weeks) rebounders. Baseline characteristics were largely comparable between those with rapid and slow rebound (Supplemental Table 4). Interestingly, the pre–treatment interruption factors associated with viral rebound differed by the timing of ART initiation (Figure 5, A and C). In early-treated participants, a higher percentage of effector memory CD8^+^ T cells and IL-6 levels were associated with slow rebound. In contrast, greater CD8^+^ T cell activation (CD38^+^HLA-DR^+^) or exhaustion (TIM3^+^), higher levels of residual viremia by the single-copy assay, and higher levels of sCD163 were associated with more rapid viral rebound (Figure 5B). For chronic-treated participants, a higher percentage of effector memory CD8^+^ T cells, HIV 1+2 total antibody (component 2), and several soluble markers (IL-21, IL-23, IL-13, IL-33) were associated with slow rebound. In contrast, rapid rebounders were marked by higher percentage of naive CD4^+^ T cells (CD45RA^+^CCR7^+^), percentage of HIV-specific CD8^+^ T cells expressing CD107a, and levels of Gal-3 and LBP (Figure 5D and Supplemental Figure 2). Of note, the IPDA was not included in this multivariable modeling due to the relatively high proportion of participants (13%) with missing values (amplification or detection failure).

## Discussion

In this analysis of the A5345 trial, we report a broad assessment of the virologic, immunologic, and inflammatory predictors of HIV rebound after treatment interruption for individuals treated during early or chronic HIV infection. The main findings of this study include the identification of (i) the far smaller and less transcriptionally active HIV reservoir in early-treated participants, (ii) immune features associated with a smaller intact proviral reservoir, (iii) predictors of HIV rebound differing between early- versus chronic-treated participants, (iv) the strongest reservoir predictor of time to HIV rebound being levels of residual viremia in early-treated participants and intact DNA levels in chronic-treated individuals, and (v) distinct sets of viral, immune, and inflammatory biomarkers before treatment interruption differentiating participants who had rapid (< 4 weeks) versus slow (≥ 4 weeks) rebound in early- and chronic-treated individuals.

A holistic characterization of the HIV reservoir involves the use of multiple assays given the complexities of the reservoir state. This includes the facts that most proviruses harbor genetic defects (e.g., mutations, deletions), rendering them unable to propagate; transcriptional activity is not limited to intact proviruses; not all genetically intact genomes can produce virions; and HIV-infected cells are relatively rare (10). Since the accurate measurement of this reservoir is crucial to guide interventions aimed at reducing the replication reservoir, many assays have been developed to characterize the HIV reservoir. This study compiled a battery of these assays, and HIV reservoir measures largely correlated well with one another, except for one notable exception: iSCA, which is designed to measure levels of residual HIV RNA in plasma (11). There is evidence that residual viremia detected by the iSCA is largely composed of clonal or oligoclonal sequences, likely emerging from the presence of highly transcriptionally active proviral clones (12, 13) that may not be easily characterized by other measures of the HIV reservoir. Similar to a previous report (14), assays that measured transcriptional activity of the reservoir (CA-RNA) showed that nonintact proviruses produced most of the measured RNA transcripts. This may be due in part to the far larger size of the defective reservoir as well as the prior observation that intact proviruses are more likely to be found in chromosomal regions of deep latency with long-term suppressive ART (15, 16). Efforts aimed at intact reservoir elimination could be led astray if only reservoir activity assays are used. As might be expected from earlier studies (5, 17, 18), we found that early-treated participants had smaller and less transcriptionally active HIV reservoirs versus chronic-treated participants. These characteristics make early-treated persons an attractive first target for curative interventions; however, most persons with HIV start ART during chronic infection and must be considered for a broadly applicable intervention.

Previous studies have evaluated a small number of specific reservoir predictors of HIV rebound timing, at times yielding disparate results. There were a few early reports suggesting that individuals with smaller total HIV DNA had delayed duration of viral rebound after ART interruption (6, 19–21), but these studies did not differentiate intact and defective proviruses. Several subsequent studies instead reported that lower active reservoir levels (i.e., lower levels of cell-associated HIV RNA and residual viremia by the single-copy assay) were also associated with delayed HIV rebound (3, 7, 22), highlighting a need for a more systematic assessment of the predictors of HIV rebound timing. One of the main goals of A5345 was to perform a more comprehensive evaluation of the reservoir, inflammatory, and immune predictors of HIV rebound timing. Our observation that the strongest reservoir measure that predicted time to HIV rebound during treatment interruption was intact (but not total) proviral DNA level in the chronic-treated group supports prior reports that the intact reservoir size could predict viral rebound timing in HIV spontaneous controllers who were discontinuing ART (23) and that extreme reservoir depletion in those who have received stem cell transplantation can lead to substantial delays in HIV rebound, even in the absence of measurable HIV-specific immune response (24). The finding that iSCA predicted the timing of HIV rebound in early- but not chronic-treated participants is intriguing, aligns with prior studies reporting the importance of the active HIV reservoir (3), and likely points to qualitative differences in the sources of the residual viremia between groups and their potential to catalyze viral rebound after ART discontinuation.

We identified immunological factors associated with a smaller HIV reservoir. Across all participants, a higher percentage of HIV Gag-specific CD8^+^ T cell cytotoxic response (CD107a) was associated with a lower IPDA. In addition, in early-treated participants, a higher percentage of CD8^+^ T cells expressing at least 2 effector cytokines was associated with lower intact proviral DNA levels. Together, this may support the hypothesis that activated CD8^+^ T cells may help clear HIV-infected cells. In contrast, HIV-specific antibody levels largely reflected the size and activity of the HIV reservoir, likely because the reservoir continues to stimulate the humoral responses (9). In human and nonhuman primate studies, the role of T cell exhaustion in predicting the timing of HIV rebound (25, 26), and our results showing that higher frequency of effector T cells and HIV-specific CD8^+^CD107a^+^ cells is associated with delayed rebound, together highlight cellular immunity as another key mediator of HIV rebound timing that deserves additional investigation (27–29). Furthermore, our results provide some of the first evidence that higher levels of LBP, sCD14, and sCD163 are associated with more rapid viral rebound, highlighting the potential influence of microbial translocation and microbiome-associated myeloid activation with the timing of HIV rebound.

Plasma markers of immune activation and inflammation might reflect immunological and inflammatory pressures on HIV rebound before and during treatment interruption. Such markers would include cytokines and chemokines (such as IFNs, IL-6, TNF-α, IL-1β), immunoregulatory circulating lectins (such as Gal-3 and Gal-9), markers of monocyte/macrophage activation (such as sCD14 and sCD163), and markers of microbial translocation (such as LBP). In our analysis, the plasma levels of sCD163 (in early-treated individuals) and sCD14 (in chronic-treated individuals) correlated with faster rebound. Plasma levels of sCD163 and sCD14 independently predict mortality during HIV infection, have been associated with the development of HIV-associated comorbidities, and have been linked to higher myeloid cell activation, especially by microbial translocation (30–34). Indeed, levels of LBP (a marker of microbial translocation) were associated with a rapid viral rebound in chronic-treated individuals. These data highlight the potential role of microbiome-associated myeloid activation during treatment interruption and suggest shared inflammatory responses between the development of HIV-associated comorbidities and HIV rebound.

Several immunomodulatory cytokines were associated with a delayed rebound in A5345 participants, including IL-6 and IL-22, in early-treated individuals. IL-6 signaling is associated with the survival and differentiation of T and B cells and the inhibition of regulatory T cell development (35–38). Notably, levels of IL-6 correlated with higher levels of effector CD8^+^ T cells and lower levels of CD8^+^ T cell exhaustion (as measured by TIM3 expression) in A5345 participants. IL-6 can also induce the development of IL-22–producing CD8^+^ T cells with enhanced effector functions (39). In our data, the plasma levels of IL-22, which correlated with a delayed rebound in early-treated participants, were associated with higher HIV-specific CD8^+^ T cell degranulation and cytokine secretion. However, it should be noted that HIV-specific CD8^+^ T cell responses have not yet been reported to be a potent mediator of HIV posttreatment control (17, 40). It is intriguing that we identified differential predictors of HIV rebound in early-treated versus chronic-treated participants. Both nonhuman primate and human studies have shown that early ART initiation represents a crucial determinant of posttreatment viral control (5, 41), and clarifying the reasons behind these observations should be of high priority. Together, our data also highlight interactions that potentially pose immunological and inflammatory pressures on HIV rebound after treatment interruption but also indicate that the factors influencing the timing of HIV rebound may diverge to some extent from the mediators of sustained posttreatment control. These potential multimodal relationships during ART and posttreatment interruption warrant further investigation.

This study had several limitations. Despite being the largest clinical trial of its kind, the study is still limited by its small number of participants in either the chronic or early group, especially the number of posttreatment controllers identified and the lack of variability in time to rebound across all participants. Given the exploratory nature of this analysis, we included *P* value adjustments only for the combined reservoir, immune, and inflammatory marker analysis, and additional studies are needed to confirm the findings here. This study also did not measure other coinfecting viruses, like human herpesviruses, that likely had some effect on HIV reservoir activity and immunologic changes (42) or epigenetic controls of proviral expression (43). Similar to most other studies in the field, we did not evaluate nonblood reservoirs, which are likely crucial in any cure effort (44). Additionally, some assays had undetectable levels (e.g., IUPM, iSCA) or missing data from assay issues (e.g., IPDA). Finally, we did not measure the size of unintegrated HIV DNA (e.g., 2–long terminal repeat circles), although it is expected that the vast majority of the HIV reservoir will be integrated in those on long-term ART (45).

Since timing of treatment impacts HIV reservoir size and dynamics and immunologic milieu (46), this study investigated 2 well-characterized groups of chronic-treated and early-treated participants. While there were certain immune phenotypes that predicted viral rebound delay in both groups, such as a higher percentage of effector memory T cells, the results catalogued largely distinct sets of viral, immune, and inflammatory biomarkers before treatment interruption that differentiated participants who had rapid versus slow viral rebound. The results provide a comprehensive overview of the complex interplay of viral, immunologic, and inflammatory predictors of viral rebound and demonstrate that the timing of ART initiation modifies the features of rapid and slow viral rebound.

## Methods

### Study design.

ACTG study A5345 was a prospective study of factors mediating the timing of HIV rebound after treatment interruption (ClinicalTrials.gov identifier: NCT03001128) (8). The study evaluated 2 cohorts of participants: individuals who initiated ART during chronic HIV infection (chronic treated, *N* = 33) or early HIV infection (early treated, *N* = 12). Individuals identified as being treated during chronic infection must have initiated ART > 6 months after the estimated date of infection, and early-treated participants must have initiated ART during Fiebig stages III–V of acute infection (47). All participants were between 18 years and 70 years of age, were on suppressive ART for ≥ 2 years with CD4^+^ count ≥ 500 cells/mm^3^ and nadir CD4^+^ count ≥ 200 cells/mm^3^, and had no history of AIDS-defining illness.

The study involved 4 steps. Step 1 included a lead-in period, such that participants on a non-nucleoside reverse transcriptase inhibitor–based regimen were switched to a protease inhibitor–based or integrase strand transfer inhibitor–based regimen (48). All participants who maintained viral suppression during step 1 were eligible to undergo treatment interruption in step 2. Participants were followed for 48 weeks or until they met the ART restart criteria, whichever occurred first. During the first 8 weeks of the treatment interruption, viral loads were monitored twice weekly by the Roche COBAS assay (Quest) and CD4^+^ cell counts every 2 weeks at local Clinical Laboratory Improvement Amendments–certified laboratories. Thereafter, viral loads were monitored weekly and CD4^+^ counts every 4 weeks. ART was restarted upon 2 successive viral loads ≥ 1,000 copies/mL, upon 1 viral load ≥ 10,000 copies/mL, or upon other predefined criteria (Supplemental Table 1). Participants who met the ART reinitiation criteria were restarted on ART (step 3) and monitored for 24 weeks after viral suppression had been achieved. Step 4 was for the long-term follow-up of individuals who were able to maintain viral control without ART.

### HIV reservoir quantification.

Samples from the time point before treatment interruption were used for reservoir, immunologic, and inflammatory marker analysis to assess for biomarker predictors of HIV rebound timing. Intracellular DNA and RNA were isolated from cryopreserved peripheral blood mononuclear cells (PBMCs) using the AllPrep DNA/RNA Mini Kit (QIAGEN). Unspliced CA-RNA and CA-DNA levels were quantified using a real-time PCR approach with primers/probes targeting conserved regions of HIV long terminal repeat (LTR)/Gag as previously described (3, 49). Cell numbers were quantified by the real-time PCR measurement of CCR5 copy numbers. Cellular integrity for RNA analysis was assessed by the measurement of total extracted RNA and evaluation of the IPO8 housekeeping gene (50). Total, intact, 3′ defective, and 5′ defective proviral reservoirs were quantified in CD4^+^ cells using the IPDA performed at AccelevirDx as described in their previous study (51). Plasma HIV residual viremia was measured using the validated ultrasensitive iSCA (limit of detection 0.56 HIV-1 RNA copies/mL) (11). Plasma from the participants was spiked with an internal replication-competent ASLV long terminal repeat with a splice acceptor virion standard as a control for RNA extraction efficiency (52). Real-time PCR reactions were performed with a Roche LightCycler 480 system using primers and probes specific to a conserved region of the HIV integrase gene (11). Finally, the number of infectious units/million resting CD4^+^ T cells (IUPM) was measured by the dQVOA as previously described (53).

### Immune phenotyping and HIV-specific immune responses.

To assess immune activation and T cell subsets, multiparameter flow cytometry was performed in batches on study entry samples of cryopreserved PBMCs from each participant. In brief, cells were stained for viability with Aqua Live/Dead (Thermo Fisher Scientific) followed by cell staining using fluorochrome-conjugated monoclonal antibodies (BD Biosciences and R&D Systems). PBMCs were stained for T cell subsets (CD3/CD4, CD3/CD8; CD3 clone UCHT1, CD4 clone RPA-T4, CD8 clone SK1) and associated markers of immune activation (CD38/HLA-DR; CD38 clone HIT2, HLA-DR clone G46-6), naive and memory T cell subsets (CD45RA/CCR7; CD45RA clone HI100, CCR7 clone 3D12), and exhaustion (PD-1, CD73, CD160, TIGIT, TIM3, LAG-3; clones EH12.1, AD2, BY55, 741182, 7D3, and T47-530, respectively). Tubes were fixed in 1% formaldehyde and analyzed within 24 hours on a BD LSRFortessa flow cytometer using BD FACSDiva software v7.0. Analysis of flow cytometry data was performed using FlowJo software (Tree Star Inc, version 9.9.3).

Pre–treatment interruption cryopreserved PBMCs were thawed and analyzed for HIV-specific polyfunctional responses. Briefly, cells were incubated with HIV peptide pools (Gag, Env, and Pol consensus clade B pool 15-mers overlapping with 11 aa; NIH Reagent Program) or PMA/ionomycin in the presence of brefeldin A, monensin, CD28/49 (all BD Biosciences), and CD107a (BioLegend) at 37°C for 6 hours followed by Aqua Live/Dead and surface staining with anti-CD3 (APC-H7, clone SK7), anti-CD4 (V450, clone RPA-T4), and anti-CD8 (PC7, clone RPA-T8) antibodies (all BD). Cells were permeabilized using 1× BD Perm/Wash buffer; incubated with anti–IFN-γ (Alexa Fluor 488), anti–IL-2 (PE, clone MQ1-14H12), and anti–TNF-α (APC, clone MAB11) antibodies (all BD); fixed; and analyzed using LSRFortessa within 24 hours. Flow cytometry data were analyzed using FlowJo v.10.8. Polyfunctional response was defined as expressing 2 or more effector cytokines following HIV peptide stimulation.

### HIV-specific antibody.

The gp41-detecting LAg avidity enzyme immunoassay (Maxim Biomedical) was performed. Assay controls and HIV-positive specimens were diluted 1:101 in specimen diluent; 100 μL of calibrator, controls, or specimens was added to antigen-coated plates; and the plates were incubated for 1 hour at 37°C. Plates were washed to remove unbound antibodies, and a dissociation buffer was added to each well for 15 minutes to release low-avidity antibodies. After further incubation with goat anti-human IgG-HRP conjugate (part of the LAg kit from Maxim Biomedical) followed by TMB substrate, plate OD was read using an absorbance plate reader (Molecular Devices Microplate Reader).

The Ortho VITROS 3600 Immunodiagnostic System (Ortho Clinical Diagnostic) was used to measure level of antibodies against HIV. Two different assays were employed, the third-generation anti-HIV 1+2 test and fourth-generation HIV combo test. The VITROS anti-HIV 1+2 test uses 4 recombinant antigens derived from HIV-1 core, HIV-1 envelope, and HIV-2 envelope. The HIV combo test is for the simultaneous qualitative detection of antibodies against HIV types 1 (including groups M and O) and 2 and HIV p24 antigen.

### Soluble markers of inflammation, immune activation, and microbial translocation.

Plasma levels of IFN-β, IFN-γ, IL-10, IL-13, IL-15, IL-1β, IL-21, IL-33, IL-6, TNF-α, fractalkine, IFN-α2a, IL-12p70, IL-2, IL-22, IL-23, IP-10, MCP-2, MIP-1α, and SDF-1a were measured by multiplex immunoassay using U-PLEX Biomarker Group 1 Assays from Meso Scale Diagnostics (catalog K15067L-2). LBP, sCD14, sCD163, galectin-3, galectin-9, and E-selectin were measured by ELISA (R&D Systems, catalog DY870-05, DY383, DY1607, DY1154, DY2045, and DY724, respectively). Plasma levels of β-glucan were measured using LAL assay (Cape Cod catalog GT003).

### Statistics.

Associations among reservoir measures, immunology markers, and time to viral rebound (first of confirmed HIV RNA values ≥ 1,000 copies/mL or first HIV RNA ≥ 10,000 copies/mL) were evaluated using the rank-based Spearman correlation. Two participants without viral rebound through 24 weeks posttreatment interruption were analyzed as the largest rank. Wilcoxon rank sum testing was performed to compare continuous variables between groups.

sPLS-DA was performed with the “mixOmics” package (version 3.15) to assess individuals with rapid (< 4 weeks) versus slow (≥ 4 weeks) viral rebound. The optimal number of components was first determined, and then the sPLS-DA model was tuned with 5-fold cross-validation 50 times, with *n* = 10 as the limitation of the maximum number of variables in each component. The optimal number of variables and number of components associated with the lowest balanced error rate were applied to the final models. VIP scores were plotted to demonstrate the contribution of selected variables to the first component. A *P* < 0.05 was considered significant. Given the exploratory nature of this study, we highlighted associations with time to viral rebound in the combined reservoir, immune, and inflammatory marker analysis by including only those associations with *P* < 0.05 and a Benjamini-Hochberg–adjusted *P* < 0.25.

### Study approval.

This study was approved by the institutional review boards of each site. All participants provided written informed consent.

### Data availability.

The authors confirm that all data underlying the figures are in the Supporting Data Values file. Additional data are available upon request from sdac.data@sdac.harvard.edu with the written agreement of the ACTG.

## Author contributions

JZL, E Aga, RJB, LF, JA, RC, JM, SD, RTG, AL, BM, and DMS designed the research study. JZL, MM, AK, MAM, ERW, JK, LBG, CDG, MP, LM, MK, LF, LB, E Acosta, JA, RB, JM, SD, RTG, MB, AL, BM, and DMS performed the study and experiments. JZL, YL, E Aga, RJB, BM, and DMS analyzed the data. ZL, MM, AK, MAM, YL, E Aga, RJB, ERW, JK, LBG, CDG, MP, LM, MK, LF, LB, E Acosta, JA, RB, JM, SD, RTG, MB, AL, BM, and DMS performed manuscript writing and editing.

## Supplementary Material

Supplemental data

Supporting data values

## Figures and Tables

**Table 1:**
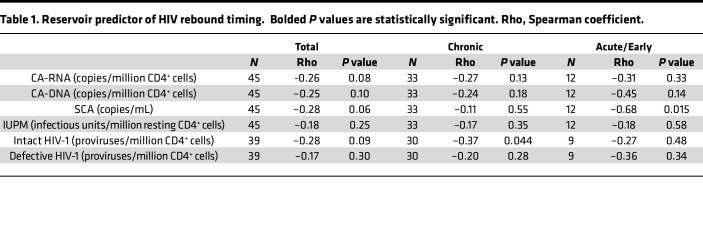
Reservoir predictor of HIV rebound timing. Bolded *P* values are statistically significant. Rho, Spearman coefficient.

## Version 1 02/08/2024

Electronic publication
