# Supplementary material for: Predictors of HIV rebound differ by timing of antiretroviral therapy initiation
Source: JCI Insight. 2024 Feb 8;9(3):e173864. doi: 10.1172/jci.insight.173864 (PMC10967395; doi:10.1172/jci.insight.173864)
Supplement: Supplemental data [file jciinsight-9-173864-s270.pdf]

Viral and Immune Predictors of HIV Rebound after ART Interruption

SUPPLEMENTAL MATERIALS

Current A5423 Team Members
CSS Representative: Elizabeth Barr
National Institutes of Health, Office of the Director, Office of Research on Women's Health

Clinical Trials Specialist: Katharine Bergstrom
ACTG Network Coordinating Center

Consultant: Jake Estes
Oregon National Primate Research Center

Consultant: Bonnie Howell
Merck Research Laboratories

DAIDS Medical Officer: Lawrence Fox
DAIDS/NIAID/NIH/HIVRB

DAIDS Protocol Pharmacist: Oladapo Alli
Pharmaceutical Affairs Branch
DAIDS, NIAID, NIH

Data Manager: Kristine Coughlin
Frontier Science & Technology Research Foundation, Inc.

Field Representative: Joan Gottesman
Vanderbilt Therapeutics (VT) CRS

Immunologist: Alan Landay
Rush University CRS

Immunologist: Cara Wilson
University of Colorado Hospital CRS

Industry Representative: Paul Zuck
Merck Research Laboratories

Investigator: Karine Dube
UNC-CH Gillings School of Global Public Health

Investigator: Lynsay MacLaren Ehui
Whitman Walker Health CRS

Investigator: Margaret Fischl
Miami Treatment and Prevention CTU

Investigator: Michael Keefer
University of Rochester HIV/AIDS CTU

Investigator (unknown if current or past)
Eugene Kroon
Thai Red Cross AIDS Research Center Treatment (TRC-ARC Treatment) CRS

Investigator: Mark Pilkinton
Vanderbilt CTU

Laboratory Data Manager (LDM): Madison Green
Frontier Science & Technology Research Foundation, Inc.

Laboratory Data Manager (LDM): Kevin Knowles
Frontier Science & Technology Research Foundation, Inc.

Laboratory Data Manager (LDM): Kyle Whitson
Frontier Science & Technology Research Foundation, Inc.

Laboratory Specialist: Rose Lagattuta
ACTG Laboratory Center at UCLA (ALC-UCLA)

Laboratory Technologist: Patricia Anthony
USC Bishop Medical Teaching and Research Maternal Child Adolescent Virology Research Lab

Pharmacologist: Courtney Fletcher
University of Nebraska Medical Center

Statistician: Evgenia Aga
Statistical and Data Analysis Center
Harvard School of Public Health

Statistician: Ronald Bosch
Statistical and Data Analysis Center
Harvard School of Public Health

Study Co-Chair: Jonathan Li
Study Co-Chair: David Smith

Study Vice-Chair: Steven Deeks
UCSF AIDS CRS
University of California San Francisco General Hospital

Study Vice-Chair: Rajesh Gandhi
Massachusetts General Hospital ACTG CRS

Virologist: John Mellors
University of Pittsburgh CTU

Previous Team Members
CSS Representative: Moses Nsubuga (Supercharger)
Joint Clinical Research Centre (JCRC)/Kampala CRS

Clinical Trials Specialist: Evelyn Hogg
ACTG Network Coordinating Center

DAIDS Medical Officer: Grace Chen
National Institute of Allergy and Infectious Diseases (NIAID)
DAIDS Medical Officer: Karin Klingman
National Institute of Allergy and Infectious Diseases
DAIDS/NIAID/NIH/HIVRB

Data Manager: Steven Bagley
Frontier Science & Technology Research Foundation, Inc.

Data Manager: Jennifer Janik
Frontier Science & Technology Research Foundation, Inc.

Data Manager: Mark Lojacono
Frontier Science & Technology Research Foundation, Inc.

Data Manager: Thomas Miller
Frontier Science & Technology Research Foundation, Inc.

Data Manager: Apsara Nair
Frontier Science & Technology Research Foundation, Inc.

Data Manager: Aimee Willett
Frontier Science & Technology Research Foundation, Inc.

Field Representative: Mary Adams
University of Rochester Adult HIV Therapeutic Strategies Network CRS

International Program Specialist: Christina Blanchard-Horan
ACTG Network Coordinating Center

International Program Specialist: Morgan Gapara
ACTG Network Coordinating Center

International Program Specialist: Nefertari Johnson
ACTG Network Coordinating Center

International Program Specialist: Ndeye (Fatou) Sow
ACTG Network Coordinating Center

Investigator: Jintanat Ananworanich
The Henry M. Jackson Foundation for the Advancement of Military Medicine

Laboratory Data Manager (LDM): Colleen Foley
Frontier Science & Technology Research Foundation, Inc.

Laboratory Data Manager (LDM): Katelyn Hergott
Frontier Science & Technology Research Foundation, Inc.

Laboratory Data Manager (LDM): Andrew Lohrum
Frontier Science & Technology Research Foundation, Inc.

Laboratory Specialist: Kim Banks
ACTG Network Coordinating Center
Laboratory Technologist: David Shugarts
University of Colorado Hospital CRS

Pharmacologist: Edward Acosta
Alabama Therapeutics CRS

Virologist: Robert Coombs
University of Washington AIDS CRS

Supplemental Table 1. ART restart criteria

HIV-1 RNA at ≥1,000 copies/mL on two consecutive measurements
A single HIV-1 RNA ≥1,000 copies/mL and an average daily HIV-1 RNA rise of ≥0.5 log_{10} copies/mL based on the most recent two viral load measurements
A single HIV-1 RNA ≥10,000 copies/mL
CD4+ T-cell count <250 cells/mm^3 on two consecutive measurements
CD4+ T-cell count <50% of the Step 1 entry CD4 count on two consecutive measurements
Clinical progression to CDC Category B or C disease
Diagnosis of Acute Retroviral Syndrome (ARS)
Pregnancy
Re-initiation of ART requested by participant or health care provider

Supplemental Table 2: Demographics for the A5345 study population.

	**Chronic-treated (N=33)**	**Early-treated (N=12)**
Age, median (Q1, Q3) years	46 (36, 53)	38 (34, 47)
Sex, % male	88%	100%
Race/ethnicity, %		
White	73%	8%
Black	12%	
Hispanic	12%	33%
Asian		50%
Other	3%	8%
Nadir CD4, cells/mm^3		
<200		
201-500	82%	83%
>500	18%	17%
Pre-ATI CD4, median (Q1, Q3) cells/mm^3	783 (651, 1028)	742 (654, 892)
Pre-ART Viral Load, median (Q1, Q3) log_{10} copies/mL	4.5 (4.0, 5.0)	5.7 (4.8, 6.3)
Duration of ART, median (Q1, Q3) years	10.3 (6.1, 15.6)	3.9 (3.1, 4.8)

Supplemental Table 3. Features included in Partial least squares-discriminant analysis (PLS-DA) models.

Features	Category	Features	Category
CA-RNA	Viral	HIV 1+2 Ab	Antibody
CA-DNA	Viral	HIV combo Ab	Antibody
SCA	Viral	LAg-Avidity	Antibody
IUPM	Viral	IFN-β	Cytokine/chemokine
CA-RNA/CA-DNA ratio	Viral	IFN-γ	Cytokine/chemokine
Baseline CD4	Cellular	IL-10	Cytokine/chemokine
Baseline CD8	Cellular	IL-13	Cytokine/chemokine
Baseline CD4/CD8 ratio	Cellular	IL-15	Cytokine/chemokine
Nadir CD4 cell count	Cellular	IL-1β	Cytokine/chemokine
%CD38+HLADR+ CD4+ T-cells	Cellular	IL-21	Cytokine/chemokine
%CD38+HLADR+ CD8+ T-cells	Cellular	IL-33	Cytokine/chemokine
%CD45RA-CCR7+ (Central Memory) CD4+ T-cells	Cellular	IL-6	Cytokine/chemokine
%CD45RA-CCR7+ (Central Memory) CD8+ T-cells	Cellular	TNF-α	Cytokine/chemokine
%CD45RA+CCR7- (Terminally Differentiated) CD4+ T-cells	Cellular	Fractalkine	Cytokine/chemokine
%CD45RA+CCR7- (Terminally Differentiated) CD8+ T-cells	Cellular	IFN-α2a	Cytokine/chemokine
%CD45RA-CCR7- (Effector Memory) CD4+ T-cells	Cellular	IL-12p70	Cytokine/chemokine
%CD45RA-CCR7- (Effector Memory) CD8+ T-cells	Cellular	IL-2	Cytokine/chemokine
%CD45RA+CCR7+ (Naive) CD4+ T-cells	Cellular	IL-22	Cytokine/chemokine
%CD45RA+CCR7+ (Naive) CD8+ T-cells	Cellular	IL-23	Cytokine/chemokine
%CD73+CD160+ CD4+ T-cells	Cellular	IP-10	Cytokine/chemokine
%CD73+CD160+ CD8+ T-cells	Cellular	MCP-2	Cytokine/chemokine
%CD160+PD1+ CD4+ T-cells	Cellular	MIP-1α	Cytokine/chemokine
%CD160+PD1+ CD8+ T-cells	Cellular	SDF-1a	Cytokine/chemokine
%CD160+ CD4+ T-cells	Cellular	LBP	Microbial translocation
%CD160+ CD8+ T-cells	Cellular	sCD14	Immune activation/ inflammation
%CD73+ CD4+ T-cells	Cellular	sCD163	Immune activation/ inflammation
%CD73+ CD8+ T-cells	Cellular	b-glucan	Microbial translocation
%LAG3+PD1+ CD4+ T-cells	Cellular	Gal-3	Immunomodulatory lectin
%LAG3+PD1+ CD8+ T-cells	Cellular	Gal-9	Immunomodulatory lectin
%LAG3+ CD4+ T-cells	Cellular	E-selectin	Immunomodulatory lectin
%LAG3+ CD8+ T-cells	Cellular		
%PD1+ CD4+ T-cells	Cellular		
%PD1+ CD8+ T-cells	Cellular		
%TIGIT+ CD4+ T-cells	Cellular		
%TIGIT+ CD8+ T-cells	Cellular		
%Tim3+ CD4+ T-cells	Cellular		
%Tim3+ CD8+ T-cells	Cellular		
%CD8 T-cells expressing CD107a (summing the 3 HIV peptides)	Cellular		
%GAG-specific CD107a+ CD8+ T-cells	Cellular		

Supplemental Table 4: Demographics for the A5345 study population categorized by individuals with rapid (<4 weeks) versus slow (⩾4 weeks) viral rebound.

	**Rapid rebound (N=29)**	**Slow rebound (N=16)**
Age, median (Q1, Q3) years	46 (35, 50)	45 (35, 54)
Sex, % male	90%	94%
Race/ethnicity, %		
White	62%	44%
Black	10%	6%
Hispanic	14%	25%
Asian	7%	25%
Other	7%	0%
Nadir CD4, cells/mm^3		
<200		
201-500	83%	81%
>500	17%	19%
Pre-ATI CD4, median (Q1, Q3) cells/mm^3	783 (651, 1028)	742 (654, 892)
Pre-ART Viral Load, median (Q1, Q3) $\log_{10}$ copies/mL	4.7 (4.0, 5.2)	4.6 (3.9, 5.8)
Duration of ART, median (Q1, Q3) years	7.1 (4.6, 12.5)	7.7 (3.8, 17.3)
Early-treated, %	21%	38%

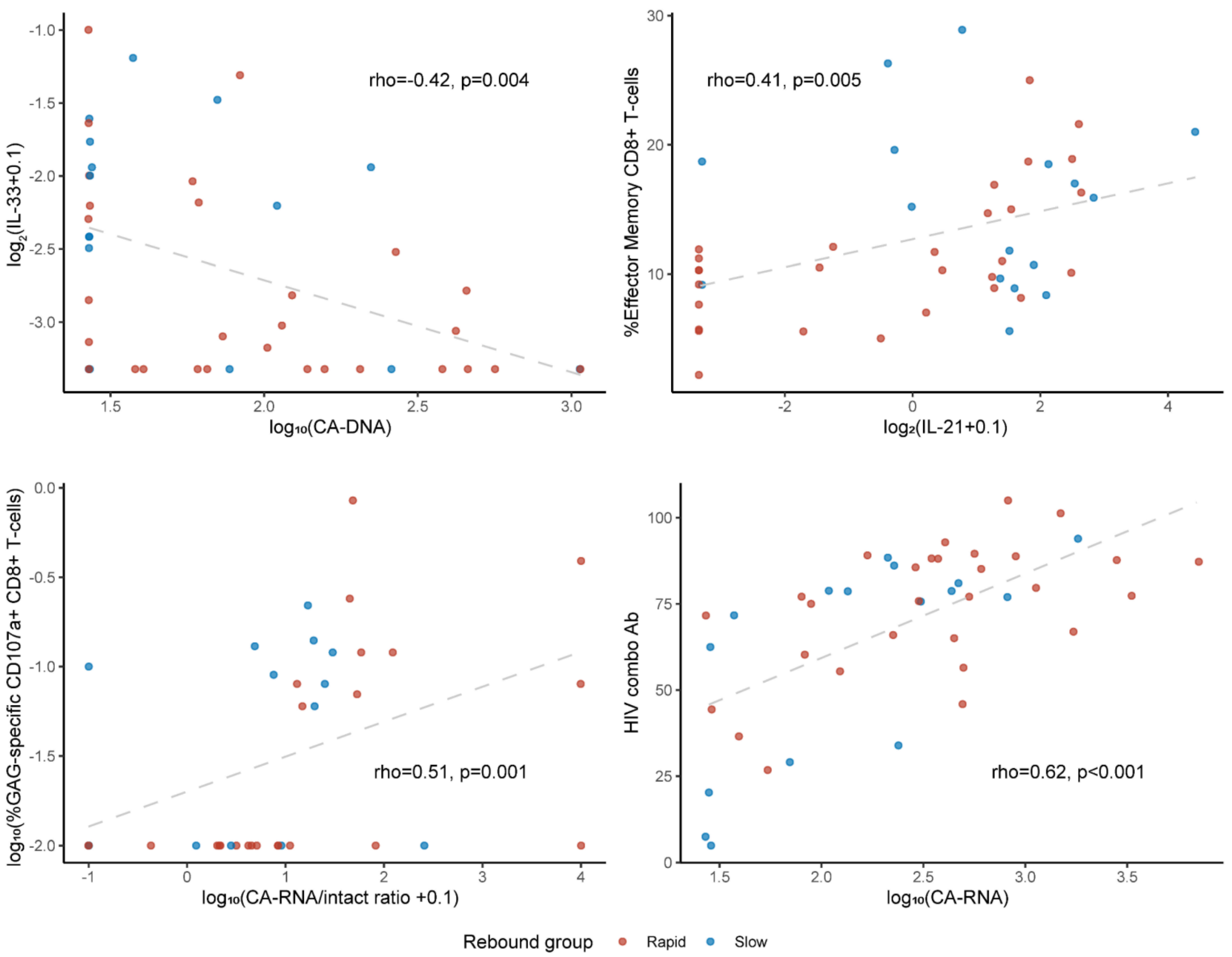

Supplemental Figure 1: Examples of significant correlations between HIV reservoir, immune and inflammatory markers. CA-DNA, total cell-associated HIV DNA; CA-RNA, unspliced cell-associated RNA. Red represents participants with HIV rebound <4 weeks and blue represents individuals with slow viral rebound ≥4 weeks.

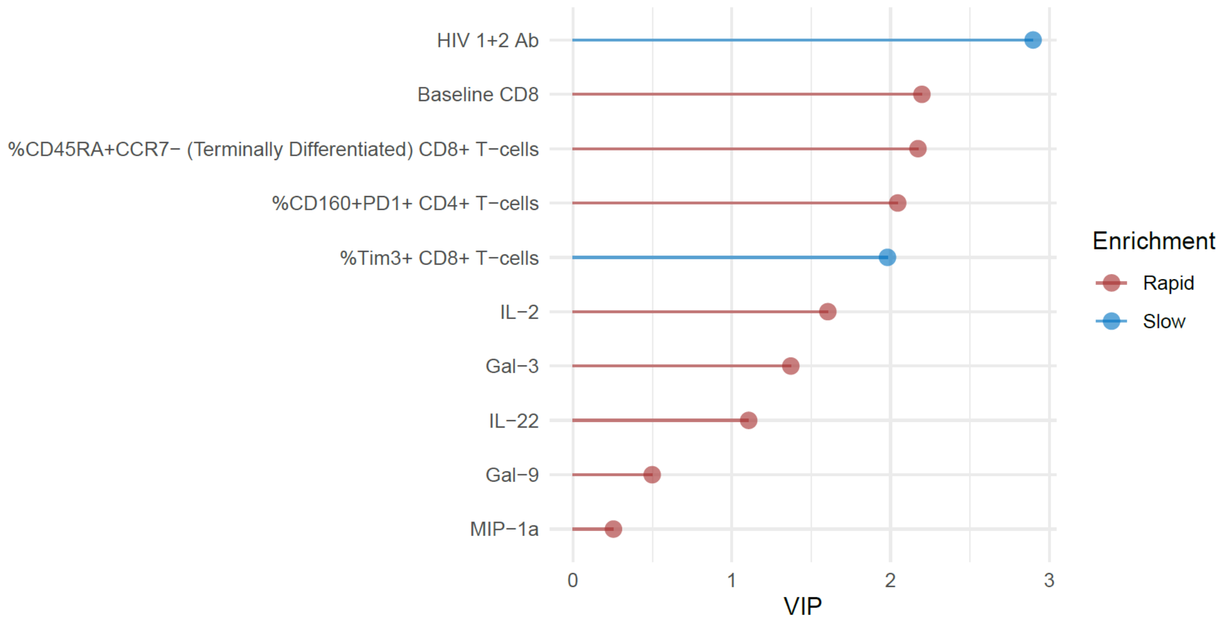

Supplemental Figure 2: Variable importance in projection (VIP) scores for selected features in the second component of chronically-treated participants.
